# Identifying Barriers to Physical Activity and Exercise Within the Bexar County Community

**DOI:** 10.7759/cureus.82387

**Published:** 2025-04-16

**Authors:** Michael D Shaffer, Virgil DeMario, Bailie Moorhead, Ressiel N Villegas, Nancy Kha, Ethan Renfrew, Michael P Naley, Niva Shrestha, Brendon McCullough, Kimberly Toumazos, Austin Smith, Arunabh Bhattacharya, Rebecca L Sanchez

**Affiliations:** 1 Department of Applied Biomedical Sciences, University of the Incarnate Word School of Osteopathic Medicine, San Antonio, USA

**Keywords:** barriers to exercise participation, community health, demographic survey, exercise equity, physical activity

## Abstract

Extensive evidence highlights the cardiovascular, metabolic, psychological, and autoimmune health benefits of physical activity (PA). Despite the well-established benefits of PA, a significant portion of the population does not meet the standard recommendations; therefore, an analysis of the various barriers to PA is worth investigating. Established barriers to PA include time, motivation, physical literacy, race, gender, and education. When one or more of these barriers exist for a population, they may significantly hinder one’s ability to perform PA. Lower levels of PA are associated with worse health outcomes. Bexar County, Texas, is one example where high rates of chronic disease are correlated with a significant lack of meeting recommended PA guidelines. An electronic survey was created to investigate the barriers to PA that exist in Bexar County. The domains surveyed were scored to help generate a total barrier score (TBS). A total of 220 adults, ages 18-99, who live in a Bexar County zip code, were surveyed. Results indicate that age was associated with decreased TBS (Estimate=-0.39, 95% CI -0.70, -0.08, p=0.015) whereas having a disability (Estimate=17.73, 95% CI 6.73, 28.73, p=0.002), having household dependents (Estimate=10.35, 95% CI 3.64, 17.05, p=0.002), and living in the northeast region (Estimate=17.6, 95% CI 2.52., 32.69, p=0.022) were associated with increased TBS. Specific barrier domains showed differences among demographics, such as increased safety barriers reported by African American individuals, decreased time barriers among older respondents, and increased health barriers among those with disabilities. By identifying and addressing barriers to PA in Bexar County, participation in local PA programs can increase, ultimately leading to improved health for the community.

## Introduction

Physical activity (PA) has consistently been shown to improve health and reduce mortality associated with various forms of chronic disease [1]. This relationship is dose-dependent, showing the greatest benefit when meeting recommendations, consistent with many international health organizations, of 150 minutes of aerobic activity and two days of large muscle group resistance training per week [1].

Regular PA, alone or coupled with energy restriction, has been shown to improve insulin sensitivity and help prevent type 2 diabetes mellitus [2]. A lack of PA is associated with up to 6% of coronary disease occurrences in the world and an increased likelihood of stroke formation [3]. Conversely, regular PA has been associated with better lipid management, reduction of atherosclerotic plaque formation, and reduction in hypertension to improve cardiovascular health [4]. Studies have demonstrated that regular PA may manage the symptoms of anxiety and depression as effectively as some pharmacological and psychological therapies [5]. Autoimmune diseases, like rheumatoid arthritis, have also been shown to benefit from PA by regulating various inflammatory pathways [6]. 

Despite the overwhelming evidence supporting the health benefits of PA, nearly half of Americans do not complete the recommended volume of PA per week [7]. Research across the globe has attempted to identify the barriers people experience that prevent them from participating in PA. Common themes across the body of research include barriers such as lack of physical literacy, social support, or enjoyment [8,9]. Other studies have identified time-related barriers like being too busy or too tired to regularly exercise [10,11]. Also, associated with occupation and work demands, people reported that their jobs require PA, so they do not participate in leisure activities [12,13,14]. In older adults, barriers such as a perceived lack of ability or fear of injury were present [15]. Several validated tools, including the Personalized Exercise Questionnaire (PEQ), Exercise Benefits/Barriers Scale (EBBS), and Centers for Disease Control (CDC) Barriers to Being Physically Active Quiz, have been utilized to measure barriers in the literature, but do not account for all potential barriers [16,17,18]. Factors such as sex, race, and education may also contribute to barriers to PA. Men are more likely to participate in weekly muscle-strengthening activities than women [19]. Healthy People 2020 showed higher rates of meeting PA recommendations in White individuals surveyed than in any other racial group [7]. College graduates have the highest rates of meeting both aerobic and strength training PA recommendations compared to those with lower levels of education [19]. Associated with education level, income can affect participation in PA, with low-income neighborhoods often lacking the critical infrastructure to support safe outdoor exercise areas [20]. 

Bexar County, Texas, comprised of San Antonio and its surrounding areas, is one of the most populous counties in the United States (U.S.) with over 2,000,000 people [21]. Over half of Bexar County residents fail to meet PA recommendations, with only 46.4% of the population reaching recommended PA levels and lower rates among Hispanic (43.9%) and Other non-Hispanic (42.7%) races, which incorporate all races that are not Hispanic, White, or Black races [21]. PA rates differ within Bexar County based on region, with the highest rates occurring in the far northeast side at 54.7% and the lowest rates on the near eastside at 39.1% [21]. The lack of adequate PA in Bexar County is likely associated with its high rates of chronic diseases such as obesity, ischemic heart disease, and diabetes [21,22]. Black and Hispanic individuals in this region showed higher rates of obesity compared to White and Other non-Hispanic individuals [21]. With such a large population of individuals failing to meet activity recommendations and high rates of chronic disease associated with a sedentary lifestyle, Bexar County and its residents could greatly benefit from research and initiatives related to increasing PA rates.

The link between regular PA and a reduction in chronic, preventable diseases cannot be ignored. Low rates of meeting PA recommendations and high rates of obesity, diabetes, and cardiovascular disease in Bexar County support this link. To our knowledge, to date, no studies have adequately identified the specific barriers to PA in Bexar County, which may differ based on race, gender, or location. This study aims to identify barriers to PA present in Bexar County, Texas, and evaluate if these barriers differ among demographics such as age, sex, race, or region of residency. By identifying and reducing barriers to PA, we may be able to increase this population’s participation in PA and thus improve their health outcomes.

## Materials and methods

The study was conducted at the University of the Incarnate Word School of Osteopathic Medicine, San Antonio. For this cross-sectional study, an electronic survey using Qualtrics (Qualtrics International Inc., Washington, USA, http://www.qualtrics.com/) was designed to investigate specific common barriers to PA in Bexar County, based on validated measures and informed by existing exercise science and health equity literature. Qualtrics was selected for its robust features related to study design, cybersecurity, and intuitive user interface. A multidisciplinary team of investigators worked closely with physical fitness experts and local practitioners, including a nutritionist, exercise psychologist, public health experts, and primary care physicians, to curate the survey questions and answers to optimize design strategy and limit survey fatigue.

Participants were recruited via social media, such as Facebook (Meta Platforms, Inc., Menlo Park, California, United States), Twitter (Twitter, Inc., San Francisco, California, United States), and Instagram (Meta Platforms, Inc., Menlo Park, California, United States), along with snowball recruitment, to reach diverse participants in Bexar County. All included respondents were required to be between the ages of 18-99 years old and live in a Bexar County-specific zip code. Potential participants were excluded if they were under the age of 18, or greater than age 99, or if they did not reside and report a Bexar County-specific zip code. The target sample size was calculated using the sample size calculation for estimating proportions calculation, which resulted in a sample size of 208 for the variables to be studied. The survey was created in English and Spanish for distribution in both languages. Data included in the analysis were collected from February 6 to March 19, 2023. Confidentiality and anonymity were strictly maintained throughout the research process. All participants accessed the survey through social media links and provided digital informed consent before participation. Survey responses did not contain personally identifiable information, and email addresses collected for incentive distribution were encrypted to ensure anonymized participant IDs. Cybersecurity was maintained utilizing a completely automated public turing test to tell computers and humans apart (CAPTCHA) prior to survey access, honeypot questions within the survey itself, and additional evaluation of response abnormalities following submission.

This study was approved by the University of the Incarnate Word Institutional Review Board (reference number 2022-1246-EX) and was conducted in accordance with the relevant ethics guidelines.

Survey measures 

The survey contained 12 demographic items, 55 barrier items, and two honeypot items for cybersecurity. Demographic information, including age, race, gender identity, sexual orientation, disability status, military service, and zip code of residence, was collected through the survey. Participants’ PA barriers were assessed using self-reporting measures across multiple domains, including environment, transportation, work, resources, safety, health, physical literacy, motivation, time, social support, and psychological factors. Respondents reported on a 4-point Likert scale, with options of strongly disagree (1), somewhat disagree (2), somewhat agree (3), and strongly agree (4), whether a listed barrier prevented them from PA. For example, “My work involved physical activity.” A full list of survey questions can be viewed in Appendix 1 (Survey Questions English Version). 

The newly developed questionnaire has not yet undergone formal validation procedures, such as exploratory or confirmatory factor analysis, to establish its psychometric properties comprehensively. However, the questionnaire was designed by adapting methodologies from validated instruments, including the PEQ, EBBS, and the CDC’s Barriers to Being Physically Active Quiz [16,17,18]. These instruments provided a framework for structuring the survey items and domains related to PA barriers.

Regarding psychometric properties, the questionnaire used a 4-point Likert scale to measure agreement with various barrier items across multiple domains: environment, transportation, work, resources, safety, health, physical literacy, motivation, time, social support, and psychological factors. The scoring system involved aggregating individual Likert scores to produce a total barrier score (TBS) and domain-specific scores for each participant. This approach is consistent with validated methods used by similar tools, which lends some preliminary credibility to the measure’s content validity.

Statistical analysis 

Statistical analysis was completed between racial groups, including African American, Asian American, Hispanic, Middle Eastern, Native American, Native Hawaiian, and White individuals. Age, region of residence (far northside, northeast, far northwest, near eastside, near northside, near westside, southeast, and southwest), sex, disability status, and household dependent status were also analyzed as key demographics. For categorical variables, a chi-squared test was performed. ANOVA was used for continuous variables. Additionally, for selected statistically significant data of interest, estimates were reported where appropriate. Univariate and multivariate regression were performed. The random forest algorithm was performed to classify significant predictors by their predictive power to generate important measures. In conjunction with clinical judgment, these important measures were used to determine the independent variables for the multivariate regression model. Power analysis was conducted to ensure an adequate sample size for detecting meaningful differences between groups. Statistical analysis was performed using R version 4.0.2 (R Foundation for Statistical Computing, Vienna, Austria). Statistical significance was accepted at a P-value < 0.05. Sensitivity analysis was performed using the subset of individuals with a higher-than-average barrier score (barrier score ≥ 110) to determine differences in the at-risk group.

## Results

This investigation delved into PA-related barriers within a diverse cohort of adult Bexar County residents, comprising African American (N=23, 10.5%), Asian American (N=33, 15%), Hispanic (N=54, 24.5%), Middle Eastern (N=4, 1.8%), Native American (N=1, 0.5%), Native Hawaiian (N=1, 0.5%), and White (N=104, 47.3%) individuals, contributing to a total cohort size of 220 as visualized in Table 1. The cohort had a mean age of 33.71 (SD 10.89), 137 (62.3%) were female, and 83 (37.7%) were male. The analysis centered on demographic characteristics and their associations with PA-related barriers. 

**Table 1:** Demographics of Bexar County participants All continuous variables were analyzed using ANOVA. Categorical variables were analyzed using the chi-square test. All other variables where appropriate underwent power analysis and Cramer's V, and degrees of freedom are listed in the table. LDS: Latter-day Saints

Demographics	African American (N=23, 10.5%)	Asian American (N=33, 15%)	Hispanic (N=54, 24.5%)	Middle Eastern (N=4, 1.8%)	Native American (N=1, 0.5%)	Native Hawaiian (N=1, 0.5%)	White (N=104, 47.3%)	Total Cohort (N=220, 100%)	Degrees of Freedom	Cramér's V	P-value
Age											
Mean	29.96 (6.68)	28.36 (7.2320)	36.46 (11.91)	30.00 (10.23)	22.00 (NA)	22.00 (NA)	35.16 (11.34)	33.71 (10.89)			0.00373
Median (Min, Max)	27.0 (20.0, 51.0)	27.0 (18.0, 60.0)	34.0 (18.0, 66.0)	26.0 (23.0, 45.0)	22.0 (22.0, 22.0)	22.0 (22.0, 22.0)	32.0 (19.0, 66.0)	31.0 (18.0, 66.0)			
Sex											
Male	15 (65.2%)	11 (33.3%)	16 (29.6%)	2 (50.0%)	1 (100%)	1 (100%)	37 (35.6%)	83 (37.7%)	6	0.243	0.044
Female	8 (34.8%)	22 (66.7%)	38 (70.4%)	2 (50.0%)	0 (0%)	0 (0%)	67 (64.4%)	137 (62.3%)			
Gender Identity											
Man	14 (60.9%)	11 (33.3%)	15 (27.8%)	2 (50.0%)	1 (100%)	1 (100%)	37 (35.6%)	81 (36.8%)	6	0.23	0.0718
Woman	9 (39.1%)	22 (66.7%)	39 (72.2%)	2 (50.0%)	0 (0%)	0 (0%)	67 (64.4%)	139 (63.2%)			
Sexual Orientation											
Asexual	0 (0%)	1 (3.0%)	1 (1.9%)	0 (0%)	0 (0%)	0 (0%)	0 (0%)	2 (0.9%)	36	0.161	0.546
Bisexual	1 (4.3%)	2 (6.1%)	2 (3.7%)	0 (0%)	0 (0%)	0 (0%)	7 (6.7%)	12 (5.5%)			
Gay/Lesbian	3 (13.0%)	1 (3.0%)	0 (0%)	1 (25.0%)	0 (0%)	0 (0%)	8 (7.7%)	13 (5.9%)			
Heterosexual	19 (82.6%)	26 (78.8%)	48 (88.9%)	3 (75.0%)	1 (100%)	1 (100%)	89 (85.6%)	187 (85.0%)			
Preferred response not listed	0 (0%)	0 (0%)	2 (3.7%)	0 (0%)	0 (0%)	0 (0%)	0 (0%)	2 (0.9%)			
Queer	0 (0%)	1 (3.0%)	1 (1.9%)	0 (0%)	0 (0%)	0 (0%)	0 (0%)	2 (0.9%)			
Questioning	0 (0%)	2 (6.1%)	0 (0%)	0 (0%)	0 (0%)	0 (0%)	0 (0%)	2 (0.9%)			
Religion											
Agnostic	0 (0%)	2 (6.1%)	2 (3.7%)	0 (0%)	0 (0%)	0 (0%)	5 (4.8%)	9 (4.1%)	132	0.357	0.0184
Atheist	1 (4.3%)	3 (9.1%)	3 (5.6%)	0 (0%)	0 (0%)	1 (100%)	5 (4.8%)	13 (5.9%)			
Baptist	2 (8.7%)	2 (6.1%)	2 (3.7%)	0 (0%)	0 (0%)	0 (0%)	2 (1.9%)	8 (3.6%)			
Buddhist	0 (0%)	2 (6.1%)	2 (3.7%)	0 (0%)	0 (0%)	0 (0%)	2 (1.9%)	6 (2.7%)			
Catholic	4 (17.4%)	6 (18.2%)	31 (57.4%)	3 (75.0%)	0 (0%)	0 (0%)	22 (21.2%)	66 (30.0%)			
Christian: Non-denominational	6 (26.1%)	4 (12.1%)	4 (7.4%)	0 (0%)	0 (0%)	0 (0%)	30 (28.8%)	44 (20.0%)			
Church of Christ	5 (21.7%)	1 (3.0%)	3 (5.6%)	0 (0%)	1 (100%)	0 (0%)	11 (10.6%)	21 (9.5%)			
Eastern Orthodox	0 (0%)	0 (0%)	0 (0%)	0 (0%)	0 (0%)	0 (0%)	1 (1.0%)	1 (0.5%)			
Episcopalian	0 (0%)	0 (0%)	0 (0%)	0 (0%)	0 (0%)	0 (0%)	2 (1.9%)	2 (0.9%)			
Hindu	1 (4.3%)	4 (12.1%)	0 (0%)	0 (0%)	0 (0%)	0 (0%)	0 (0%)	5 (2.3%)			
Jewish: Orthodox	0 (0%)	0 (0%)	0 (0%)	0 (0%)	0 (0%)	0 (0%)	1 (1.0%)	1 (0.5%)			
Jewish: Other	0 (0%)	0 (0%)	0 (0%)	0 (0%)	0 (0%)	0 (0%)	1 (1.0%)	1 (0.5%)			
LDS (Mormon)	0 (0%)	1 (3.0%)	0 (0%)	0 (0%)	0 (0%)	0 (0%)	1 (1.0%)	2 (0.9%)			
Lutheran	0 (0%)	0 (0%)	0 (0%)	0 (0%)	0 (0%)	0 (0%)	2 (1.9%)	2 (0.9%)			
Methodist	0 (0%)	0 (0%)	1 (1.9%)	0 (0%)	0 (0%)	0 (0%)	8 (7.7%)	9 (4.1%)			
Muslim	0 (0%)	6 (18.2%)	0 (0%)	1 (25.0%)	0 (0%)	0 (0%)	0 (0%)	7 (3.2%)			
None	3 (13.0%)	1 (3.0%)	4 (7.4%)	0 (0%)	0 (0%)	0 (0%)	8 (7.7%)	16 (7.3%)			
Other	0 (0%)	0 (0%)	1 (1.9%)	0 (0%)	0 (0%)	0 (0%)	0 (0%)	1 (0.5%)			
Pentecostal	0 (0%)	0 (0%)	1 (1.9%)	0 (0%)	0 (0%)	0 (0%)	0 (0%)	1 (0.5%)			
Presbyterian	0 (0%)	0 (0%)	0 (0%)	0 (0%)	0 (0%)	0 (0%)	2 (1.9%)	2 (0.9%)			
Protestant: Non-denominational	0 (0%)	0 (0%)	0 (0%)	0 (0%)	0 (0%)	0 (0%)	1 (1.0%)	1 (0.5%)			
Seventh Day Adventist	1 (4.3%)	0 (0%)	0 (0%)	0 (0%)	0 (0%)	0 (0%)	0 (0%)	1 (0.5%)			
Taoist	0 (0%)	1 (3.0%)	0 (0%)	0 (0%)	0 (0%)	0 (0%)	0 (0%)	1 (0.5%)			
Disability											
No	20 (87.0%)	33 (100%)	45 (83.3%)	4 (100%)	0 (0%)	1 (100%)	96 (92.3%)	199 (90.5%)	6	0.281	0.00793
Yes	3 (13.0%)	0 (0%)	9 (16.7%)	0 (0%)	1 (100%)	0 (0%)	8 (7.7%)	21 (9.5%)			
Service in the U.S. Armed Forces, Military Reserves, or National Guard											
Currently serving	2 (8.7%)	3 (9.1%)	5 (9.3%)	0 (0%)	0 (0%)	0 (0%)	9 (8.7%)	19 (8.6%)	12	0.158	0.528
No longer serving	3 (13.0%)	0 (0%)	9 (16.7%)	0 (0%)	0 (0%)	0 (0%)	6 (5.8%)	18 (8.2%)			
Never served	18 (78.3%)	30 (90.9%)	40 (74.1%)	4 (100%)	1 (100%)	1 (100%)	89 (85.6%)	183 (83.2%)			
Dependents											
No	10 (43.5%)	28 (84.8%)	33 (61.1%)	3 (75.0%)	1 (100%)	0 (0%)	56 (53.8%)	131 (59.5%)	6	0.263	0.0185
Yes	13 (56.5%)	5 (15.2%)	21 (38.9%)	1 (25.0%)	0 (0%)	1 (100%)	48 (46.2%)	89 (40.5%)			
Region of Residence											
Far northside	2 (8.7%)	6 (18.2%)	12 (22.2%)	0 (0%)	0 (0%)	0 (0%)	26 (25.0%)	46 (20.9%)	42	0.22	0.0157
Far northwest	2 (8.7%)	3 (9.1%)	5 (9.3%)	0 (0%)	0 (0%)	1 (100%)	11 (10.6%)	22 (10.0%)			
Near eastside	2 (8.7%)	0 (0%)	9 (16.7%)	0 (0%)	0 (0%)	0 (0%)	11 (10.6%)	22 (10.0%)			
Near northside	8 (34.8%)	5 (15.2%)	11 (20.4%)	0 (0%)	0 (0%)	0 (0%)	26 (25.0%)	50 (22.7%)			
Near westside	3 (13.0%)	11 (33.3%)	6 (11.1%)	2 (50.0%)	1 (100%)	0 (0%)	13 (12.5%)	36 (16.4%)			
Northeast	2 (8.7%)	5 (15.2%)	2 (3.7%)	2 (50.0%)	0 (0%)	0 (0%)	4 (3.8%)	15 (6.8%)			
Southeast	4 (17.4%)	3 (9.1%)	5 (9.3%)	0 (0%)	0 (0%)	0 (0%)	5 (4.8%)	17 (7.7%)			
Southwest	0 (0%)	0 (0%)	4 (7.4%)	0 (0%)	0 (0%)	0 (0%)	8 (7.7%)	12 (5.5%)			

Based on an a priori power analysis, a sample size of 185 was required to achieve 80% power at an alpha level of 0.05 for detecting a small effect size. Our final sample of 220 participants met these criteria, ensuring adequate power for statistical analyses. 

Age distribution 

The cohort age was notably young with an overall cohort mean of 33.705 years (SD=10.888). This variability in age was observed across demographic groups (ANOVA, p=0.00373). 

Sex and gender disparities 

Sex distribution exhibited significant differences (chi-square test, p=0.044) with a predominantly female cohort. Participant-reported gender did not significantly vary. Among specific racial groups, males constituted a higher percentage in the African American (n=15, 65.2%) and White (n=37, 35.6%) groups, while females were more prevalent in the Asian American (n=22, 66.7%) and Hispanic (n=38, 70.4%) groups. Gender distribution displayed a trend towards significance (chi-square test, p=0.0718), with a higher percentage of individuals identifying as men in the African American (n=14, 60.9%) and White (n=37, 35.6%) groups, and women being more represented in the Asian American (n=22, 66.7%) and Hispanic (n=39, 72.2%) groups.

Sexual orientation composition 

No significant differences were observed in sexual orientation across demographic groups (chi-square test, p=0.546). Heterosexuality represented the largest proportion of participants, ranging from 75.0% (n=3) in the Middle Eastern group to 88.9% (n=48) in the Hispanic group, with an overall prevalence of 85.0% (n=187). 

Religious affiliation 

Statistical significance was noted in religious affiliation (chi-square test, p=0.0184), revealing variations among groups. Catholicism was more prevalent in the Hispanic (n=31, 57.4%) and White (n=22, 21.2%) groups. 

Disability status 

A significant difference was found between disability status and demographic groups (chi-square test, p=0.00793), with a prevalence of 9.5% (n=21) of the total cohort identifying as a person with disability. 

Military service 

No significant differences were observed in military service status across demographic groups (chi-square test, p=0.528), with most participants having never served. 

Household dependents 

Significant differences were found between household dependents and demographic groups (chi-square test, p=0.0185), with prevalence of those who had household dependents representing 40.5% (n=89) of the total sample. 

Regional disparities 

Significant differences were found between regions and racial groups (chi-square test, p=0.0157). The near northside showed the largest proportion of African American respondents at 34.8% (n=8) of the demographic. The near westside represented the largest proportion of Asian American respondents, with 33.3% (n=11) residing in this region. The largest proportion of Hispanic individuals identified themselves as living in the far northside with 22.2% (n=12). The two regions with the largest proportion of White individuals were the near northside and the far northside, representing 25% (n=26) of the White respondents, respectively. 

Total barrier score (TBS)

The multivariate regression analysis provided insights into the relationship between demographic variables and barrier scores to PA while controlling for age, race, sex, region, disability, and dependents, as demonstrated in Table 2. Age emerged as a significant predictor, with older individuals exhibiting lower TBS (Estimate=-0.39, 95% CI -0.70, -0.08, p=0.015). While no statistically significant differences were observed among racial groups or between male and female sex, regional differences were evident, with individuals residing in the northeast (Estimate=17.6, 95% CI 2.52, 32.69, p=0.022) region reporting higher TBS compared to those from the far northside. This underscores the influence of environmental and contextual factors on perceived barriers to PA. Moreover, participants with reported disabilities (Estimate=17.73, 95% CI 6.73, 28.73, p=0.002) and dependents (Estimate=10.35, 95% CI 3.64, 17.05, p=0.002) exhibited significantly elevated barrier scores, highlighting the complex interplay between personal circumstances and exercise accessibility. TBS also did not differ significantly by race, region, sex, dependents, or disability in a subgroup analysis of those with above-average barrier scores (barrier score > 110). However, age was still negatively associated with TBS in this subgroup analysis (Estimate=-0.40, p=0.008).

**Table 2:** Multivariate linear regression of barriers to physical activity Highlighted/bolded values in the tables represent statistical significance of p <0.05.

Multivariate Analysis			
Demographics	Estimate	(95% CI)	P-value
Age	-0.39	-0.70 – -0.08	0.015
Race			
White	Ref		
African American	7.16	-3.52 – 17.83	0.189
Asian American	4.48	-5.16 – 14.12	0.363
Hispanic	3.77	-3.78 – 11.32	0.328
Middle Eastern	9.1	-14.59 – 32.79	0.451
Native American	31.43	-15.22 – 78.07	0.187
Native Hawaiian	-1.42	-47.23 – 44.40	0.952
Sex			
Male	Ref		
Female	-2.04	-8.79 – 4.70	0.553
Region			
Far northside	Ref		
Far northwest	9.05	-2.84 – 20.94	0.136
Near eastside	-2.64	-14.65 – 9.37	0.667
Near northside	-2.64	-14.65 – 9.37	0.667
Near westside	3.83	-6.70 – 14.36	0.476
Northeast	17.6	2.52 – 32.69	0.022
Southeast	-0.53	-14.86 – 13.79	0.942
Southwest	7.31	-5.73 – 20.36	0.272
Disability			
No, I do not have a disability	Ref		
Yes, I have a disability	17.73	6.73 – 28.73	0.002
Dependents			
No	Ref		
Yes	10.35	3.64 – 17.05	0.002

Barrier domain scores 

Specific barrier domains were also analyzed to identify which barrier types were significant among each demographic group accounted for in the regression. Significant barrier domains were identified for age, race, sex, region of residence, disability status, and dependents in the household status. A full review of multivariate linear regression statistics for each barrier domain can be seen in Tables 3-6. Environmental barriers were significantly increased for those with disabilities (Estimate=1.1, 95% CI 0.33, 1.87, p=0.005), but not for any other demographics. Transportation-related barriers were decreased for Middle Eastern respondents (Estimate=0.77, 95% CI 0.06, 1.48, p=0.033), residents of the near northside (Estimate=0.3, 95% CI 0.01, 0.59, p=0.04), and those with dependents in the house (Estimate=0.38, 95% CI 0.18, 0.58, p=<0.001) and increased for only Native American respondents (Estimate=2.61, 95% CI 1.21, 4.01, p=<0.001). Work-related barriers were significantly decreased with age (Estimate=-0.02, 95% CI -0.04, -0.00 p=0.046), African American race (Estimate=0.8, 95% CI 0.19, 1.40, p=0.01), female sex (Estimate=-0.46, 95% CI -0.84, -0.08, p=0.019), and those with household dependents (Estimate=0.59, 95% CI 0.21, 0.97, p=0.02). Resource-related barriers showed regional dependence as they increased in the northeast (Estimate=3.55, 95% CI 1.22, 5.88, p=0.003), near northside (Estimate=2.41, 95% CI 0.93, 3.89, p=0.001), and southwest (Estimate=2.03, 95% CI 0.02, 4.05, p=0.048) regions compared to the far northside. Additionally, those with disabilities (Estimate=2.78, 95% CI 1.09, 4.48, p=0.001) and with household dependents (Estimate=1.62, 95% CI 0.59, 2.66, p=0.002) also showed an increased barrier score in the resource domain.

**Table 3:** Environmental barrier domain multivariate linear regression Highlighted/bolded values in the tables represent statistical significance of p <0.05.

Multivariate Analysis			
Demographics	Estimate	(95% CI)	P-value
Age	-0.01	-0.03 – 0.01	0.515
Race			
White	Ref		
African American	0.43	-0.32 – 1.18	0.265
Asian American	0.41	-0.27 – 1.08	0.239
Hispanic	-0.01	-0.54 – 0.52	0.958
Middle Eastern	0.2	-1.46 – 1.86	0.812
Native American	2.7	-0.57 – 5.98	0.106
Native Hawaiian	-0.57	-3.78 – 2.65	0.73
Sex			
Male	Ref		
Female	0.28	-0.19 – 0.76	0.24
Region			
Far northside	Ref		
Northeast	0.08	-0.97 – 1.14	0.876
Far northwest	0.05	-0.79 – 0.88	0.909
Near eastside	-0.02	-0.87 – 0.82	0.955
Near northside	0.05	-0.62 – 0.72	0.885
Near westside	-0.31	-1.05 – 0.42	0.405
Southeast	-0.79	-1.79 – 0.22	0.125
Southwest	-0.71	-1.63 – 0.21	0.129
Disability			
No, I do not have a disability	Ref		
Yes, I have a disability	1.1	0.33 – 1.87	0.005
Dependents			
No	Ref		
Yes	0	-0.47 – 0.47	0.989

**Table 4:** Transportation barrier domain multivariate linear regression Highlighted/bolded values in the tables represent statistical significance of p <0.05.

Multivariate Analysis			
Demographics	Estimate	(95% CI)	P-value
Age	-0.01	-0.02 – 0.00	0.073
Race			
White	Ref		
African American	0.19	-0.13 – 0.51	0.251
Asian American	0.13	-0.16 – 0.42	0.381
Hispanic	0.08	-0.15 – 0.30	0.507
Middle Eastern	0.77	0.06 – 1.48	0.033
Native American	2.61	1.21 – 4.01	<0.001
Native Hawaiian	0.2	-1.17 – 1.57	0.773
Sex			
Male	Ref		
Female	-0.15	-0.35 – 0.05	0.151
Region			
Far northside	Ref		
Northeast	0.37	-0.08 – 0.83	0.104
Far northwest	0.23	-0.12 – 0.59	0.203
Near eastside	0.17	-0.19 – 0.53	0.346
Near northside	0.3	0.01 – 0.59	0.04
Near westside	-0.02	-0.34 – 0.30	0.903
Southeast	0.19	-0.24 – 0.62	0.378
Southwest	0.24	-0.15 – 0.63	0.229
Disability			
No, I do not have a disability	Ref		
Yes, I have a disability	0.22	-0.11 – 0.55	0.185
Dependents			
No	Ref		
Yes	0.38	0.18 – 0.58	<0.001

**Table 5:** Work barrier domain multivariate linear regression Highlighted/bolded values in the tables represent statistical significance of p <0.05.

Multivariate Analysis			
Demographics	Estimate	(95% CI)	P-value
Age	-0.02	-0.04 – -0.00	0.046
Race			
White	Ref		
African American	0.8	0.19 – 1.40	0.01
Asian American	-0.45	-1.00 – 0.10	0.109
Hispanic	-0.32	-0.75 – 0.11	0.14
Middle Eastern	0.03	-1.32 – 1.37	0.97
Native American	-1.89	-4.54 – 0.76	0.161
Native Hawaiian	0.5	-2.11 – 3.10	0.709
Sex			
Male	Ref		
Female	-0.46	-0.84 – -0.08	0.019
Region			
Far northside	Ref		
Northeast	0.41	-0.45 – 1.27	0.347
Far northwest	0.21	-0.46 – 0.89	0.536
Near eastside	0.28	-0.40 – 0.97	0.416
Near northside	0.29	-0.26 – 0.83	0.303
Near westside	0.3	-0.30 – 0.90	0.321
Southeast	0.19	-0.62 – 1.01	0.641
Southwest	0.4	-0.34 – 1.14	0.287
Disability			
No, I do not have a disability	Ref		
Yes, I have a disability	-0.11	-0.74 – 0.51	0.729
Dependents			
No	Ref		
Yes	0.59	0.21 – 0.97	0.002

**Table 6:** Resource barrier domain multivariate linear regression Highlighted/bolded values in the tables represent statistical significance of p <0.05.

Multivariate Analysis			
Demographics	Estimate	(95% CI)	P-value
Age	-0.01	-0.06 – 0.03	0.551
Race			
White	Ref		
African American	0.34	-1.30 – 1.99	0.682
Asian American	1.32	-0.16 – 2.81	0.081
Hispanic	0.31	-0.85 – 1.48	0.598
Middle Eastern	-0.05	-3.71 – 3.60	0.977
Native American	2.15	-5.05 – 9.35	0.558
Native Hawaiian	0.27	-6.80 – 7.34	0.941
Sex			
Male	Ref		
Female	-1.02	-2.06 – 0.02	0.055
Region			
Far northside	Ref		
Northeast	3.55	1.22 – 5.88	0.003
Far northwest	1.64	-0.20 – 3.47	0.08
Near eastside	0.55	-1.30 – 2.41	0.56
Near northside	2.41	0.93 – 3.89	0.001
Near westside	1.59	-0.03 – 3.22	0.055
Southeast	1.51	-0.70 – 3.73	0.179
Southwest	2.03	0.02 – 4.05	0.048
Disability			
No, I do not have a disability	Ref		
Yes, I have a disability	2.78	1.09 – 4.48	0.001
Dependents			
No	Ref		
Yes	1.62	0.59 – 2.66	0.002

Age was found to be associated with decreasing safety barrier score (Estimate=-0.14, 95% CI -0.22, -0.07, p=<0.001), while African American race (Estimate=4.2, 95% CI 1.60, 6.80, p=0.002), Asian American race (Estimate=2.37, 95% CI 0.03, 4.72, p=0.047), northeast residence (Estimate=4.8, 95% CI 1.13, 8.47, p=0.01), far northwest residence (Estimate=3.14, 95% CI 0.24, 6.03, p=0.034), and near northside residence (Estimate=2.98, 95% CI 0.64, 5.31, p=0.012) showed increased safety barriers. Physical literacy-related barriers were found to be decreased in Hispanic respondents (Estimate=0.92, 95% CI 0.15, 1.68, p=0.019) and increased in Native American respondents (Estimate=6.12, 95% CI 1.39, 10.85, p=0.011), and residents in the northeast (Estimate=1.85, 95% CI 0.32, 3.38, p=0.018) and near northside (Estimate=1.01, 95% CI 0.03, 1.98, p=0.042). Motivation-related barriers were found to be increased for those with disabilities (Estimate=4.45, 95% CI 1.13, 7.76, p=0.009) and those with dependents (Estimate=2.41, 95% CI 0.39, 4.43, p=0.02) only (Tables 7-9).

**Table 7:** Safety barrier domain multivariate linear regression Highlighted/bolded values in the tables represent statistical significance of p <0.05.

Multivariate Analysis			
Demographics	Estimate	(95% CI)	P-value
Age	-0.14	-0.22 – -0.07	<0.001
Race			
White	Ref		
African American	4.2	1.60 – 6.80	0.002
Asian American	2.37	0.03 – 4.72	0.047
Hispanic	1.44	-0.40 – 3.28	0.124
Middle Eastern	2.26	-3.50 – 8.03	0.442
Native American	5.62	-5.73 – 16.97	0.332
Native Hawaiian	5.88	-5.27 – 17.02	0.301
Sex			
Male	Ref		
Female	1.38	-0.26 – 3.03	0.098
Region			
Far northside	Ref		
Northeast	4.8	1.13 – 8.47	0.01
Far northwest	3.14	0.24 – 6.03	0.034
Near eastside	0.9	-2.02 – 3.83	0.544
Near northside	2.98	0.64 – 5.31	0.012
Near westside	2.36	-0.20 – 4.92	0.071
Southeast	1.34	-2.15 – 4.82	0.452
Southwest	1.81	-1.36 – 4.99	0.263
Disability			
No, I do not have a disability	Ref		
Yes, I have a disability	2.25	-0.43 – 4.92	0.1
Dependents			
No	Ref		
Yes	1.21	-0.42 – 2.84	0.145

**Table 8:** Physical literacy barrier domain multivariate linear regression Highlighted/bolded values in the tables represent statistical significance of p <0.05.

Multivariate Analysis			
Demographics	Estimate	(95% CI)	P-value
Age	-0.02	-0.05 – 0.01	0.239
Race			
White	Ref		
African American	-0.11	-1.19 – 0.98	0.848
Asian American	0.33	-0.65 – 1.31	0.509
Hispanic	0.92	0.15 – 1.68	0.019
Middle Eastern	2.29	-0.11 – 4.69	0.062
Native American	6.12	1.39 – 10.85	0.011
Native Hawaiian	-2.41	-7.06 – 2.23	0.309
Sex			
Male	Ref		
Female	-0.4	-1.08 – 0.29	0.257
Region			
Far northside	Ref		
Northeast	1.85	0.32 – 3.38	0.018
Far northwest	0.32	-0.89 – 1.53	0.602
Near eastside	-0.74	-1.96 – 0.48	0.236
Near northside	1.01	0.03 – 1.98	0.042
Near westside	-0.17	-1.24 – 0.90	0.757
Southeast	0	-1.45 – 1.45	1
Southwest	1.14	-0.19 – 2.46	0.092
Disability			
No, I do not have a disability	Ref		
Yes, I have a disability	0.56	-0.56 – 1.67	0.33
Dependents			
No	Ref		
Yes	0.6	-0.08 – 1.28	0.084

**Table 9:** Motivation barrier domain multivariate linear regression Highlighted/bolded values in the tables represent statistical significance of p <0.05.

Multivariate Analysis			
Demographics	Estimate	(95% CI)	P-value
Age	-0.07	-0.17 – 0.02	0.133
Race			
White	Ref		
African American	0.7	-2.52 – 3.92	0.671
Asian American	-0.71	-3.61 – 2.20	0.633
Hispanic	0.04	-2.24 – 2.31	0.975
Middle Eastern	-0.46	-7.60 – 6.68	0.899
Native American	6.91	-7.16 – 20.97	0.336
Native Hawaiian	-3.52	-17.34 – 10.29	0.617
Sex			
Male	Ref		
Female	-0.26	-2.30 – 1.77	0.8
Region			
Far northside	Ref		
Northeast	3.32	-1.23 – 7.87	0.152
Far northwest	2.09	-1.49 – 5.67	0.253
Neareast	-1.79	-5.41 – 1.83	0.332
Near northside	2.02	-0.87 – 4.92	0.17
Near westside	0.62	-2.55 – 3.80	0.701
Southeast	-1.05	-5.37 – 3.27	0.635
Southwest	1.09	-2.84 – 5.02	0.587
Disability			
No, I do not have a disability	Ref		
Yes, I have a disability	4.45	1.13 – 7.76	0.009
Dependents			
No	Ref		
Yes	2.41	0.39 – 4.43	0.02

The health barrier domain was found to be decreased in female respondents (Estimate=-1.21, 95% CI -1.88, -0.53, p<0.001) and increased in those with a disability (Estimate=2.84, 95% CI 1.75, 3.94, p<0.001). Time-specific barriers were decreased with age (Estimate=-0.06, 95% CI -0.11, -0.01, p=0.01) and increased in those with dependents (Estimate=1.65, 95% CI 0.65, 2.66, p=0.001) with no significant race, sex, or regional differences. Social support barriers were not found to be significantly different for any of the demographic groups. Finally, psychological barriers were found to be increased in those with disabilities (Estimate=1.68, 95% CI 0.55, 2.81, p=0.004) and decreased in those with household dependents (Estimate=0.83, 95% CI 0.14, 1.52, p=0.018) and with age (Estimate=-0.06, 95% CI -0.09, -0.03, p=<0.001). Significant differences in specific barrier domains inform which barriers contribute most to significant TBS. They also help identify how interventions can best be tailored for different regions of Bexar County or among different demographics (Tables 10-13). 

**Table 10:** Health barrier domain multivariate linear regression Highlighted/bolded values in the tables represent statistical significance of p <0.05.

Multivariate Analysis			
Demographics	Estimate	(95% CI)	P-value
Age	-0.01	-0.04 – 0.03	0.716
Race			
White	Ref		
African American	0.08	-0.98 – 1.15	0.876
Asian American	0.35	-0.61 – 1.31	0.48
Hispanic	0.26	-0.50 – 1.01	0.506
Middle Eastern	-0.53	-2.89 – 1.84	0.663
Native American	4.07	-0.58 – 8.72	0.086
Native Hawaiian	1.17	-3.39 – 5.74	0.614
Sex			
Male	Ref		
Female	-1.21	-1.88 – -0.53	<0.001
Region			
Far northside	Ref		
Northeast	0.24	-1.27 – 1.74	0.756
Far northwest	0.24	-0.94 – 1.43	0.688
Neareast	0	-1.20 – 1.20	0.999
Near northside	0.93	-0.03 – 1.88	0.057
Near westside	-0.07	-1.12 – 0.98	0.894
Southeast	-0.61	-2.04 – 0.82	0.404
Southwest	1.15	-0.15 – 2.45	0.084
Disability			
No, I do not have a disability	Ref		
Yes, I have a disability	2.84	1.75 – 3.94	<0.001
Dependents			
No	Ref		
Yes	0.43	-0.24 – 1.09	0.212

**Table 11:** Time barrier domain multivariate linear regression Highlighted/bolded values in the tables represent statistical significance of p <0.05.

Multivariate Analysis			
Demographics	Estimate	(95% CI)	P-value
Age	-0.06	-0.11 – -0.01	0.01
Race			
White	Ref		
African American	0.94	-0.66 – 2.54	0.25
Asian American	1	-0.45 – 2.44	0.175
Hispanic	0.11	-1.02 – 1.24	0.853
Middle Eastern	1.87	-1.68 – 5.42	0.301
Native American	-0.49	-7.47 – 6.50	0.892
Native Hawaiian	-1.07	-7.93 – 5.79	0.76
Sex			
Male	Ref		
Female	0.09	-0.92 – 1.10	0.86
Region			
Far northside	Ref		
Northeast	1.64	-0.26 – 3.54	0.091
Far northwest	0.59	-0.90 – 2.09	0.438
Neareast	-0.99	-2.50 – 0.52	0.199
Near northside	1.19	-0.02 – 2.40	0.054
Near westside	0.6	-0.73 – 1.92	0.379
Southeast	0.41	-1.39 – 2.21	0.656
Southwest	0.49	-1.15 – 2.13	0.56
Disability			
No, I do not have a disability	Ref		
Yes, I have a disability	0.71	-0.94 – 2.35	0.4
Dependents			
No	Ref		
Yes	1.65	0.65 – 2.66	0.001

**Table 12:** Social support barrier domain multivariate linear regression

Multivariate Analysis			
Demographics	Estimate	(95% CI)	P-value
Age	0.02	-0.02 – 0.06	0.324
Race			
White	Ref		
African American	-0.23	-1.58 – 1.11	0.734
Asian American	0.17	-1.04 – 1.39	0.778
Hispanic	0.61	-0.34 – 1.56	0.207
Middle Eastern	0.3	-2.68 – 3.29	0.842
Native American	1.64	-4.23 – 7.51	0.584
Native Hawaiian	-0.72	-6.49 – 5.05	0.807
Sex			
Male	Ref		
Female	-0.06	-0.75 – 0.64	0.872
Region			
Far northside	Ref		
Northeast	1.64	-0.26 – 3.54	0.091
Far northwest	0.59	-0.90 – 2.09	0.438
Neareast	-0.99	-2.50 – 0.52	0.199
Near northside	1.19	-0.02 – 2.40	0.054
Near westside	0.6	-0.73 – 1.92	0.379
Southeast	0.41	-1.39 – 2.21	0.656
Southwest	0.49	-1.15 – 2.13	0.56
Disability			
No, I do not have a disability	Ref		
Yes, I have a disability	1.25	-0.13 – 2.64	0.076
Dependents			
No	Ref		
Yes	0.62	-0.23 – 1.46	0.151

**Table 13:** Psychological barrier domain multivariate linear regression Highlighted/bolded values in the tables represent statistical significance of p <0.05.

Multivariate Analysis			
Demographics	Estimate	(95% CI)	P-value
Age	-0.06	-0.09 – -0.03	<0.001
Race			
White	Ref		
African American	-0.18	-1.28 – 0.92	0.749
Asian American	-0.45	-1.44 – 0.54	0.376
Hispanic	0.35	-0.43 – 1.13	0.377
Middle Eastern	2.42	-0.02 – 4.85	0.052
Native American	1.98	-2.81 – 6.77	0.417
Native Hawaiian	-1.14	-5.85 – 3.56	0.634
Sex			
Male	Ref		
Female	-0.06	-0.75 – 0.64	0.872
Region			
Far northside	Ref		
Northeast	0.48	-1.07 – 2.03	0.546
Far northwest	0.1	-1.12 – 1.33	0.867
Neareast	-0.64	-1.87 – 0.60	0.312
Near northside	0.88	-0.10 – 1.87	0.079
Near westside	0.13	-0.95 – 1.21	0.815
Southeast	-0.8	-2.27 – 0.67	0.288
Southwest	-0.28	-1.62 – 1.06	0.683
Disability			
No, I do not have a disability	Ref		
Yes, I have a disability	1.68	0.55 – 2.81	0.004
Dependents			
No	Ref		
Yes	0.83	0.14 – 1.52	0.018

## Discussion

**Figure 1:**
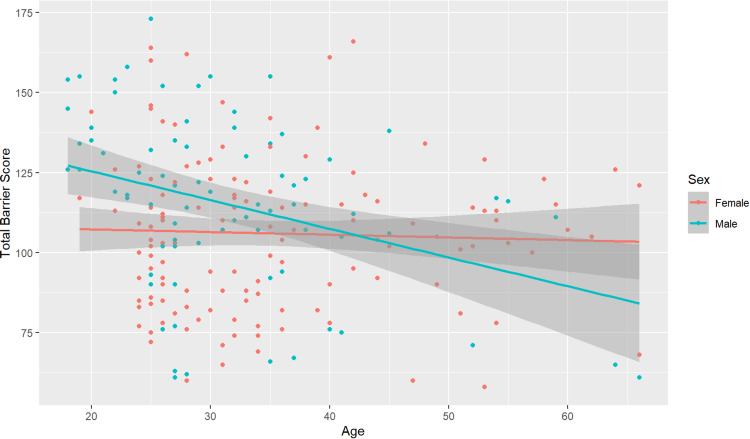
Total barrier score by age and sex

This study controlled for region, race, age, disability status, household dependents, and sex when identifying barriers to PA. When evaluating TBS, age, disability status, and household dependents were associated with significantly different barriers. Older age was associated with decreased TBS, as seen in Figure 1, suggesting that younger participants consistently identified more barriers than older participants. Previous literature consistently elucidated that time is a significant barrier to PA across many demographic groups. According to one study, the top two perceived barriers reported were a lack of time, cited by 65.3% of participants, followed closely by feelings of tiredness at 64.7% [23]. Another study shed light on the pervasive nature of the time constraint challenge, suggesting that perceiving other obligations and responsibilities as more significant and time-consuming deterred individuals from participating in PA [24]. Age in this study was also associated with decreased work, time, and psychological barrier domains. Though specifics regarding time spent working or caring for dependents in the household were not gathered in this study, it is possible that older participants in the study had more time to exercise or perform PA compared to their young counterparts, accounting in part for the decreased TBS. Those who reported having dependents conversely reported an increased barrier score in the time domain. Community exercise groups geared towards family-friendly PA could help address this barrier.

**Figure 2:**
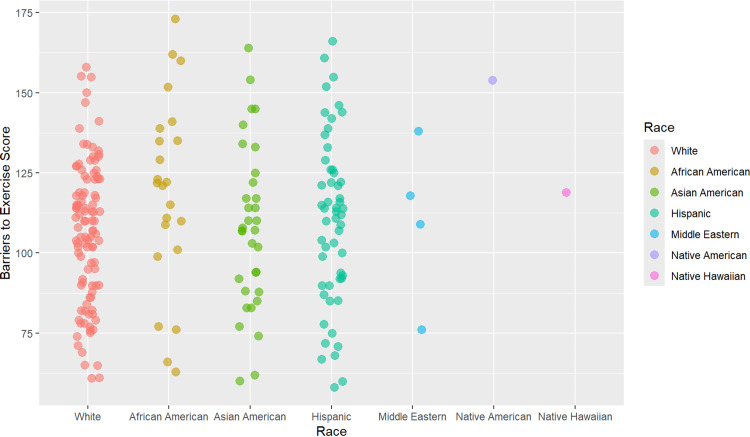
Total barrier score by race

TBS was not significant between races, as shown in Figure 2, but specific domains were identified to be significant for different races. Safety was a significant barrier for African American and Asian American respondents, showing an increased barrier score compared to the White race. The built environment, or the physical makeup of an environment, including homes, schools, sidewalks, and transportation, among other infrastructure, can significantly influence PA levels in a community [25]. Respondents in the study reported that feeling unsafe when exercising outdoors was a barrier to activity. This may be due to the built environment, crime-related safety concerns present in different communities in Bexar County, or examples of nationally covered events that influence the perception of safety [20,26,27]. The near northside, near eastside, and far northwest regions of Bexar County showed increased safety barriers compared to the far northside. According to crime statistics, these regions contain more zip codes with violent crime rates of 2,400 or higher per 100,000 people compared to the zip codes in the far northside [21]. The near northside region, home to the highest proportion of African American respondents in the study, has many high-crime zip codes, including 78212, 78216, and 78217 [20]. Perceptions of safety for various demographics are likely related to having experienced discrimination in similar spaces. For example, one study showed that Black men were less likely to exercise in neighborhoods that were perceived as predominantly White neighborhoods [28]. Safety concerns are complex and multifaceted, but interventions aimed at increasing safety, such as police presence and practice, may best address this barrier for African American individuals in Bexar County. Additionally, community exercise groups, such as running clubs, may try to develop a presence in these areas to promote feelings of safety while exercising in a group. 

This study found that Hispanic individuals reported a significantly decreased barrier score to physical literacy. On the other hand, Native American individuals and individuals who live in the northside and northeast regions of Bexar County reported an increased barrier score in physical literacy. Physical literacy is an all-encompassing concept describing the various characteristics of an individual that allow them to participate in PA, such as competence, affect, understanding, and physical skills for movement [29].

Research has shown that having high or moderate physical literacy levels during late adolescence can significantly impact overall PA and fitness levels [30]. Older adults with inadequate physical literacy were shown to be 38% less likely to have exercised in the past five days than those with adequate physical literacy [31]. Observational studies have found positive relationships between PA and educational attainment, suggesting that physical literacy education could be a key factor in increasing PA in a population [32]. Despite this, most U.S. states only require minimal or unspecified amounts of physical education time across elementary, middle, and high school education [33]. Coupled with poor funding, inadequate facilities, and large class sizes, physical education and thus physical literacy are lacking across the country, including within Bexar County [33]. However, the existing body of literature in this field remains limited, highlighting the need to further explore and understand the relationship between physical literacy and sustained engagement in PA among adolescents and adults. By delving deeper into this area, researchers can uncover valuable insights that can inform strategies to promote physical literacy and overall PA. The San Antonio Fitness in the Park courses help develop physical literacy through guided fitness classes, but could be specifically geared toward developing independent PA programming and techniques. These strategies may be best implemented in the near northside and northeast regions of Bexar County, where this barrier was identified as significant. 

The survey identified that health-related barriers were increased in those with disabilities. A previous study highlighted the significant impact of current injuries or disabilities as barriers to increased PA, revealing independent associations with age and body mass index even after accounting for other variables [34]. This finding underscores the importance of tailoring PA promotions, especially for older adults, those with disabilities, and individuals struggling with weight issues, by focusing on safe activities that do not exacerbate existing injuries or disabilities [34]. A study evaluating PA barriers in women with arthritis found that pain, stiffness, and limitations were common arthritis-specific barriers among participants in addition to general barriers such as fatigue, motivation, and absence of social support [10]. Despite the numerous benefits of moderate PA for arthritis, these barriers exist and highlight the need for tailored interventions to address challenges specific to chronic health conditions and disability [10]. This study noted that respondents with disabilities also reported increased barrier scores related to environmental, resource, motivation, and psychological domains, suggesting the complex nature of PA barriers to those with disabilities. Other local organizations that support those with disabilities in the county may also utilize this information to consider exercise classes or groups and exercise programming literature as part of their offered resources.

Individuals who perceived fewer barriers to exercise were more likely to participate in PA programs than those who perceived more barriers [35]. Community-based exercise programs aimed at targeting identified barriers could be an effective strategy to reduce barriers to participation in exercise since community-based exercise programs are effective in promoting exercise adherence [36,37]. A study evaluating community-based programs, including walking groups and PA classes, suggested that these programs improved exercise adherence and overall PA levels in older adults [37]. A key recommendation of a qualitative systematic review regarding implementing a community exercise program is to anticipate and identify the barriers to exercise in the community in which the program is being implemented [38]. These findings suggest the importance of social support in fostering exercise adherence and that community-based exercise programs can provide a supportive environment and enhance social networks, leading to fewer perceived barriers and increased exercise adherence. Community leaders and exercise groups can help to target barriers by promoting experiences geared toward specific groups with common identities, such as women’s groups, parent groups, or disability groups with modified PA programs. Respondents participating in this study received a brochure with a compiled set of resources, including PA recommendations, basics of exercise programming, nutrition information, and a map of City of San Antonio Fitness in the Park locations to promote physical literacy and help respondents identify community-based PA opportunities. 

By identifying barriers to exercise in Bexar County, Texas, community-based exercise groups can be better informed about how to meet the needs of the communities they serve. This should promote more successful implementation of community-based exercise programs across Bexar County. Reducing identified barriers will make exercise more equitable for Bexar County residents, and social support provided by these programs should improve exercise participation and adherence among individuals in these communities. This will allow more people across the county to participate in and experience the health benefits of exercise, leading to a healthier Bexar County.

Strengths and limitations 

The survey was easily accessible to participants across Bexar County, requiring only Internet access to complete. Additionally, our survey was offered in both English and Spanish. According to Bexar County reports, 84.4% of households have access to the Internet, and 88.7% of people report speaking English very well [21]. Though not all adults in Bexar County utilize social media, the combination of social media and snowball recruitment methods appears to have reached a geographically diverse portion of the population. Some level of sampling bias, based on utilizing an online survey and predominantly web-based social media recruitment, is present in our study. However, this survey was accessible to most adults living in Bexar County based on these methodological characteristics.

A novel instrument was developed for this study to assess traditionally evaluated barriers alongside potential demographic barriers such as those related to race, gender, or sexual identity. Development of the survey was performed by a culturally diverse multidisciplinary team. The diverse backgrounds of the survey developers, consultation with experts in nutrition, public health, exercise psychology, and medicine, and a thorough review of the literature to develop the survey resulted in a holistic evaluation of exercise barriers. A variety of validated survey measures were evaluated in the creation of our research tools, such as the CDC’s Barriers to Being Physically Active Quiz, the EBBS, and PEQ [16,17,18]. None of these fully captured the domains of interest specific to Bexar County; thus, a new tool was developed. Specific barriers that other tools did not account for included those related to race, religion, and sexuality, which were assessed by our survey tool. Barrier domains like these tools and the strategy of aggregating domain scores and total scores were utilized in the development of our research tool. As discussed in the study methods, though no formal validation process was utilized prior to dispersing our survey, the aforementioned factors contribute to the validity of the novel tool.

Limitations in this study included not gathering direct socioeconomic status data and the potential for illegitimate response collection. Socioeconomic status can be extrapolated from zip code data as mean household income or education level based on Bexar County demographic reports; however, neither of these items was collected specifically [21]. Additionally, with widespread social media recruitment and a cash incentive for participation, cybersecurity was a consideration in this study. Despite appropriate measures being taken, upon completion of data collection, not all surveys collected were utilized in the final analysis. Illegitimate responses were identified and manually excluded from the analysis using data validation tools and methodology. Finally, with a self-report survey utilized to gather data, response bias was present. No data was specifically collected on the amount of PA performed; rather, each participant’s perceived barriers required self-reporting. There is no completely objective measure to evaluate an item such as a perceived barrier, so the reliability of self-report data can be questioned. Specific diseases, such as diabetes, heart disease, or rheumatologic conditions, were also not evaluated by our tool; rather, if a respondent identified as having a disability or not. Disability type was collected but not utilized in the analysis of the data. Future studies may utilize more objective measures of PA, like accelerometers or evaluation of specific diseases, like diabetes, coupled with perceived barriers, to identify the correlation between noted barriers and actual PA participation. Despite these limitations, the study provides valuable insights into the exercise barriers experienced by the diverse Bexar County community.

## Conclusions

This study identified that barriers to PA in Bexar County, Texas, differ based on age, race, disability status, household dependents, and region of residence. Identifying significant barriers to PA is a crucial first step to addressing each community's specific needs to successfully elevate PA rates. Many of the barriers discussed warrant further investigation to elicit more precise solutions, such as tailored community-based exercise programs, increasing safety in neighborhoods, and increasing physical literacy. Future solutions will help bridge the gap of inequities present in current exercise participation and facilitate the holistic improvement of health in Bexar County.

## Appendices

Appendix 1

Survey Questions English Version

Demographics: 

What is your current age (in years)? 

(Numeric Response 18-99)

What is your current sex? 

Male 

Female 

Preferred response not listed (Please specify):________ 

What is your gender/gender identity? 

Man 

Woman 

Transgender man 

Transgender woman 

Gender Queer (Non-binary) 

Gender non-conforming  

Preferred response not listed (Please specify): ________ 

What is your sexual orientation? 

Heterosexual 

Bisexual 

Gay/Lesbian 

Queer 

Questioning 

Asexual 

Preferred response not listed (Please specify): ________ 

Please indicate the racial or ethnic groups with which you identify (Check all that apply.) 

African American

Asian American

Hispanic

Middle Eastern/North African 

Native American/Alaskan Native 

Native Hawaiian/Other Pacific Islander 

White  

Other (Please specify): ________ 

Asked of any person indicating that they identify with more than one racial or ethnic group.

Of the following, please mark the ONE racial or ethnic group with which you most identify. 

African American

Asian American

Hispanic

Middle Eastern/North African 

Native American/Alaskan Native 

Native Hawaiian/Other Pacific Islander 

White  

Other 

With what religious background, if any, do you most identify? 

Agnostic 

Atheist 

Bahá’í 

Baptist 

Buddhist 

Catholic 

Church of Christ 

Christian: Non-Denominational 

Confucian 

Eastern Orthodox 

Episcopalian 

Hindu 

Muslim 

Jehovah’s Witness 

Jewish: Orthodox 

Jewish: Other 

LDS (Mormon) 

Lutheran 

Methodist 

Pentecostal 

Presbyterian 

Protestant: Non-Denominational 

Quaker 

Seventh Day Adventist 

Taoist 

Unitarian/Universalist 

UCC/Congregational 

None 

Other (Please specify): ________ 

Do you have a disability? 

Yes, I have a disability  

No, I do not have a disability 

What type(s) of disabilities do you have? (Check all that apply.) 

Acquired/traumatic brain injury 

Attention deficit/hyperactivity disorder 

Asperger's/Autism spectrum 

Blind/low vision 

Deaf/hard of hearing 

Cognitive or learning disability 

Chronic illness/medical condition 

Mental health/psychological condition 

Physical/mobility condition that affects walking 

Physical/mobility condition that does not affect walking 

Speech/communication condition 

Other (Please specify): ________ 

Have you ever served in the U.S. Armed Forces, Military Reserves, or National Guard? 

I am currently serving   

I am no longer serving 

I have never served 

Do you have dependents in the household? 

Yes 

No 

In what zip code do you reside?  

Enter via the drop-down menu for zip codes within Bexar County 

Read the following prompts and select the most applicable response.  

Strongly agree (4), Somewhat agree (3), Somewhat disagree (2), Strongly disagree (1) 

Environmental 

(1) The weather prevents me from exercising (too hot/cold or wet/rainy) 

(2) My allergies prevent me from exercising  

Transportation 

(3) I do not have transportation to get to an exercise site on my own  

Work 

(4) My work involves physical activity 

(5) I commute to work by walking or riding my bike, so I do not feel the need to exercise outside of that 

Resources 

(6) There are no gyms or exercise facilities near me 

(7) If we had exercise facilities and showers at work, then I would be more likely to exercise 

(8) I cannot afford a gym membership or classes 

(9) I don’t have home exercise equipment (free weights, yoga mats, exercise bands, machines) 

(10) I don’t have reliable internet to access online exercise resources, videos, and programs 

(11) My community lacks adequate outdoor spaces to exercise (jogging trails, swimming pools, bike paths, sidewalks, parks, heavy traffic, etc.) 

(12) My community lacks adequate indoor spaces to exercise (community centers, schools, gyms, etc.)  

(13) I don’t have access to adequate childcare, or I cannot afford childcare resources, so I don’t exercise  

Safety 

(14) I feel unsafe when exercising outdoors due to my gender  

(15) I feel unsafe when exercising outdoors due to my race  

(16) I feel unsafe when exercising outdoors due to the lack of lights (it is dark outside) 

(17) I feel unsafe when exercising outdoors due to safety concerns in my neighborhood  

(18) I feel unsafe when exercising outdoors due to my sexual orientation 

(19) I feel unsafe when exercising at a gym due to my gender  

(20) I feel unsafe when exercising at a gym due to my race 

(21) I feel unsafe when exercising at a gym due to my sexual orientation 

(22) I am afraid of injuring myself while exercising 

(23) I avoid public exercise due to fear of exposure to COVID or other diseases 

(24) I do not have a safe place to exercise (e.g., proper place to exercise, dry and clean floors, good lighting, etc.) 

Exercise Literacy 

(25) I don’t know how to properly and safely exercise 

(26) I don’t know how to generate an exercise plan 

(27) I don’t know where to find information on how to exercise 

Personal/Motivation 

(28) I don’t enjoy exercising 

(29) I don’t feel motivated to exercise for any reason 

(30) I don’t feel the need to exercise because it does not affect my health 

(31) I am too tired before or after work to get any exercise 

(32) I don’t get enough sleep as it is, and I just couldn’t get up early or stay up late to get some exercise

(33) I’ve been thinking about getting more exercise, but I just can’t seem to get started 

(34) I don’t get enough exercise because I have never learned the skills for any sport

(35) I want to get more exercise, but I just can’t seem to make myself stick to anything

(36) Physical activity is just not fun

(37) I really can’t see learning a new sport at my age

(38) It’s easier for me to find excuses not to exercise than to go out to do something

(39) I do not exercise because of religious reasons

Health 

(40) I am not healthy enough to exercise 

(41) I have a disability that prevents me from exercising 

(42) Exercise may worsen one or more health conditions I have  

(43) My diet affects my ability to exercise  

Time 

(44) I don’t have time to exercise around my work schedule  

(45) Physical activity takes too much time away from other commitments: time, work, family, etc. 

(46) My free time during the days/weekends is too short to include exercise

(47) I don’t have time to exercise because I must care for my family or members of my household 

(48) Exercise is not a priority for me during my free time 

Social Support 

(49) No one in my life encourages me to exercise 

(50) I do not have anyone to exercise with 

(51) None of my family members or friends like to do anything active, so I don’t have a chance to exercise

(52) My usual social activities with family or friends do not include physical activity

Psychological 

(53) I do not like how my body looks while exercising 

(54) I feel like I’m using equipment that would be better off used by someone else  

(55) I am afraid of being judged by others while exercising in public 

Please select the answer choice “blue” below: 

Red 

Green 

Blue 

Purple 

Please select all options below that are a color: 

Red 

Potato 

Yellow 

Apple 
